# Pre-diagnosis recreational physical activity and lung cancer survival within the California teachers study

**DOI:** 10.1007/s10552-026-02169-6

**Published:** 2026-04-22

**Authors:** Emily L. Cauble, Mia Blanchard, Peggy Reynolds, Emma S. Spielfogel, Jessica Clague DeHart

**Affiliations:** 1 City of Hope, Beckman Research Institute, Division of Health Analytics, Duarte, CA USA; 2 School of Community and Global Health, Claremont Graduate University, Claremont, CA USA; 3 Department of Epidemiology and Biostatistics, University of California San Francisco, Berkeley, CA USA

**Keywords:** Lung cancer, Survivorship, Physical activity, Risk factors, Cohort study

## Abstract

**Purpose:**

Although physical activity (PA) levels have been linked to decreased lung cancer mortality, the magnitude of associations and delineation of biological and behavioral risk factors are often inconsistent. Our study aims to address this gap by elucidating the associations of lung cancer survival while considering duration and intensity of prediagnosis PA levels.

**Methods:**

We evaluated lung cancer-specific survival among 1768 women diagnosed with lung cancer in the California Teachers Study (1995–2019). PA was assessed at baseline and included lifetime PA (cumulative activity from high school through age 54) and recent PA (activity during the 3 years prior to enrollment), each quantified separately for moderate and strenuous intensity. These measures capture both long-term duration and short-term duration of PA at different intensities before diagnosis. Multivariable Cox proportional hazards models using age as the time scale were used to estimate hazard ratios (HRs) and 95% confidence intervals (CIs) for lung cancer-specific mortality.

**Results:**

Similar risks of lung cancer-specific mortality were observed across most PA variables. Among ever and/or former smokers, higher levels of moderate, lifetime PA were associated with lower lung cancer mortality (HR_ever_ = 0.77; HR_former_ = 0.63). In contrast, ever and/or current smokers who engaged in intermediate to high levels of strenuous, lifetime PA had higher mortality risk (intermediate: HR_ever_ = 1.24; HR_former_ = 1.39). Among never smokers, higher strenuous, lifetime PA was associated with lower mortality risk (HR = 0.69, 95% CI = 0.49–0.97), while intermediate levels of strenuous, recent PA were associated with higher mortality (HR = 1.62, 95% CI = 1.12–2.34).

**Conclusion:**

The results of this study suggest that smoking significantly modifies the association between physical activity and lung cancer‑specific survival. The mechanisms underlying these patterns remain uncertain, and additional research is needed to clarify how physical activity duration and intensity relate to lung cancer prognosis across smoking histories.

**Supplementary Information:**

The online version contains supplementary material available at 10.1007/s10552-026-02169-6.

## Introduction

According to the American Cancer Society, lung cancer is the 2nd most common cancer in women in the USA, and the leading cause of cancer death [1]. Although incidence and mortality rates have decreased over time due to advancements in treatment and early detection, the number of lung cancer-related deaths remains substantial; women have also been shown to have smaller decreases in incidence compared to men (8% vs 14% decrease) [1–4]. In 2024, it was estimated that lung cancer will account for over 125,000 deaths in the USA [1–3].

The most common form of lung cancer, non-small cell lung cancer (NSCLC), is often diagnosed at an advanced stage [1, 5]. NSCLC accounts for 80–85% of lung cancer cases, and even with treatment, has a 5-year survival rate of only 28%; specifically, women have a 31.3% 5-year survival estimate [6, 7]. The 5-year survival rate for small cell lung cancer is even lower at 7% [6]. Successful treatment depends on disease severity, and while systemic therapy modestly prolongs survival in patients with advanced lung cancer, tumors in some patients are highly resistant to therapy [5, 8]. This grave picture highlights the crucial necessity to investigate biological mechanisms and identify factors that may be used to develop interventions, tailor treatment regimens, and improve lung cancer prognosis.

Smoking is widely recognized as a primary risk factor for lung cancer development and progression for both men and women, with a substantial body of literature reporting that smokers have an increased risk for developing and dying from lung cancer compared to non-smokers [9, 10]. Research examining the biological mechanisms of this association has demonstrated that smokers have elevated levels of various immune markers (e.g., increased white blood cell counts and pro-inflammatory markers), as well as increased oxidative stress compared to non-smokers [11]. Smoking promotes chronic, systemic inflammation by directly impacting epithelial and immune cells within the airway (via the oral and nasal cavities), releasing various pro-inflammatory immune markers and activating additional immune cells [9, 12].

To help prevent lung cancer development and improve prognoses, healthier lifestyle choices are often encouraged, including physical activity (PA) and smoking cessation. Although PA levels have been linked to decreased lung cancer risk and mortality in previous epidemiological studies, these studies differ greatly in the magnitude of significant associations and inconsistently delineate by biological sex at birth, smoking status, PA levels, and lifetime PA leading up to a lung cancer diagnosis [13–22]. Our study aims to further elucidate the associations of lung cancer mortality with time-varying and exertion-varying PA levels and to address the inconsistent associations presented in previous epidemiological studies.

## Methods

### Study population and data collection

The California Teacher Study (CTS) cohort was established in 1995–1996 and consists of 133,477 active and retired female teachers and administrators in California. The cohort has been previously described [23], and participants provided informed consent at baseline. This project was approved by the Institutional Review Board of Claremont Graduate University. All methods were executed in accordance with relevant institutional and national guidelines. Due to standard cohort exclusions (i.e., consenting to breast cancer research only, moving out of California before completion of the baseline survey, invalid baseline surveys due to missing data, participant death before the return of the baseline survey, person(s) other than an identified proxy completing the baseline survey, or a follow-up questionnaire was completed prior to the baseline survey), the starting eligible population for this cohort study was 125,120 women. The study start date was the date that each participant completed the baseline questionnaire (late 1990’s). The following additional exclusions were applied to arrive at a final analytical cohort: participants whose start age was the same as their end age were censored (no follow-up) (*n* = 64), lung cancer incidence before the start date (*n* = 188), incomplete smoke exposure data (*n* = 841), lung cancer was not classified as malignant (invasive) in both ICD-O-3 and ICD-O-2 (*n* = 6), incomplete PA data (*n* = 857), and incomplete alcohol consumption data (n = 6,061). A total of 1,768 participants (including 516 never smokers) who were diagnosed with lung cancer between the time of joining the cohort and the end of 2019 were deemed eligible and are included in our analyses. Of the eligible cohort, 1,043 women died of lung cancer during the study period.

At the start of the study follow-up period, participants submitted a baseline questionnaire that covered extensive demographic and personal information, including PA levels (lifetime PA [from high school through age 54 years] and recent PA [in the three years before joining the cohort]), recent and past hormonal therapy use, menopausal status, and smoking status/exposure. The CTS cohort is linked annually with the California Cancer Registry (CCR) and the California Department of Public Health (CDPH) to ascertain cancer diagnoses and tumor information, as well as date and cause of death in cohort members, respectively.

## Participant covariates

Covariates collected at baseline and considered for our analysis have been associated with lung cancer mortality in previous studies [15, 16, 19]. These covariates include age, race/ethnicity (Non-Hispanic White and Other), first-degree family history of lung cancer (parent, sibling, or child: yes, no, and adopted/not provided), Body Mass Index (BMI) calculated from collected weight and height variables (BMI; < 18 kg/m^2^, 18–24 kg/m^2^, 25–29 kg/m^2^, ≥ 30 kg/m^2^), education level (less than high school, technical/high school diploma, associate degree/some college, and university or higher [graduated]), and alcohol consumption (none, < 20 g/day, or ≥ 20 g/day). Menopausal status (premenopausal, perimenopausal, and postmenopausal) was collected at baseline and derived from responses about menstrual periods; additional data were collected for duration and timing of estrogen and progestin therapy and ages at reported reproductive organ surgeries, if relevant. Only baseline covariates were available for all participants in the study cohort. As such, all covariate data were collected at the baseline questionnaire, prior to lung cancer diagnosis.

Participants also provided detailed information regarding active and passive smoking history. Respondents were asked if they had ever smoked 100 or more cigarettes during their lifetime and, if so, when they started and stopped smoking. Information on smoking history was also collected, including total lifetime smoking pack-years, the presence of household passive smoke exposure, and years since quitting for former smokers. A derived smoking variable was generated that incorporated smoking status and total pack-years and was defined as the following levels: never smokers (no pack-years), former smokers who had low pack-years (≤ median pack-years for former smokers), former smokers who had high pack-years (> median pack-years for former smokers), current smokers who had low pack-years (≤ median pack-years for current smokers), and current smokers who had high pack-years (> median pack-years for current smokers). The median pack-years for former smokers was 22.05, and the median pack-years for current smokers was 38.25. This variable was used for adjustment in the main regression models to account for smoking dose and cumulative exposure.

## Physical activity variables

Participants provided detailed information on the baseline questionnaire regarding recreational PA across various periods of their lives (while in high school; between the ages of 18 and 24, 25 and 34, 35 and 44, and 45 and 54 years, as well as during the 3 years before completing the questionnaire) and before lung cancer diagnosis. For each time interval, they were asked to indicate the average amount of time spent participating in all moderate activities (e.g., brisk walking, recreational tennis, volleyball, golf, softball, and cycling on level street) and in all strenuous activities (e.g., swimming laps, aerobics, calisthenics, running, jogging, cycling on hills, and racquetball). Participants reported the average number of hours per week (categories: none, 0.5, 1, 1.5, 2, 3, 4–6, 7–10, and ≥ 11 h) and months per year (categories: 1–3, 4–6, 7–9, and 10–12 months) they engaged in moderate and strenuous PA. For each time interval, separate “hours per week” variables were created for strenuous and moderate PA by multiplying the hours spent per week by the portion of the year in which the woman engaged in the activity.

Lifetime PA was calculated for each participant by multiplying the average hours per week per year (h/wk/y) of activity performed during one of the time periods by the number of years of the relevant time interval and then summing across all time periods. The cumulative measure was divided by the total number of years spent in all the time periods to provide an average annual lifetime (beginning with high school through current age if < 55 years at baseline) or quasi-average annual lifetime (if 55 years or older at baseline) measure of PA for each woman. A woman’s PA during the three years before completing the baseline questionnaire (recent activity) was also assessed. Each PA variable (moderate and strenuous for lifetime and recent activity) was categorized into tertiles based on distribution cut points for further analysis (descriptive statistics presented in Supplemental Table S1). Additionally, a combined PA variable (moderate + strenuous activity, presented as tertiles) was created for both lifetime and recent activity (Supplemental Table S1).

## Statistical analyses

Descriptive analyses were conducted to characterize the study population. Multivariable-adjusted hazard ratios (HR) and 95% confidence intervals (CI) for lung cancer mortality associated with PA were obtained by fitting Cox proportional hazards regression models using age as a timescale (where subjects enter at the age they were diagnosed with lung cancer and exit at their event/censoring age). Using age as the time metric ensures that women of the same age are compared and, therefore, controls for differences in the risk of death due to age alone. The first analytical model (Model 1) included adjusting only for the respective PA variables as appropriate (e.g., when assessing the mortality risk for moderate lifetime PA, the model was adjusted for age at diagnosis and strenuous lifetime PA). This approach allows for the association for the specific PA variable to be assessed without any confounding effects from the other PA variables (e.g., strenuous activity can be assessed irrespective of the impact of moderate activity). The second model (Model 2) expanded upon Model 1, with additional adjustments for menopause status with hormonal therapy use, smoking total pack-years by smoking status, and alcohol consumption. Sensitivity analyses were performed for additional covariates of interest, including BMI, education level, passive smoke exposure, smoking quit-years, and disease stage. Additionally, detailed treatment data were not available; however, since lung cancer treatment is based greatly on tumor stage, stage at diagnosis was used as a proxy in our statistical models.

Follow-up time was calculated as the number of days between the lung cancer diagnosis and the date of death, the date the participant moved out of California, or the end of study date (31 December 2019), whichever came first. Women who moved out of California, died from a cause other than lung cancer, or did not have an event before 31 December 2019, were censored and contributed person-days to the analysis only up to the date of the respective event.

To examine whether the association between physical activity and lung cancer mortality differed by smoking history, we conducted stratified analyses within four conventional smoking categories: never, ever, former, and current [13, 15, 16]. These HRs reflect separate models run within each smoking group. “Ever smokers” represent the combined former and current groups. This categorization was selected for interpretability and to ensure adequate sample size within strata. Kaplan–Meier curves and log-rank tests were used to examine the differences in survival by level of PA and were based on time since diagnosis, not age at diagnosis, as this is the conventional and clinically intuitive metric for visualizing survival after a cancer diagnosis [15]. All statistical analyses were performed using SAS® software, version 9.4 [24] and were performed in the secure CTS platform [25].

## Results

The mean follow-up period from baseline to the date of diagnosis was 15 years, and the median time from baseline to lung cancer mortality was 13.2 years. The average age at diagnosis was 74.7 (± 9.9) years (Table 1). The majority of women were diagnosed with NSCLC (93.2%) and nearly half presented with distant metastases (49.7%). premenopausal women were more likely to have higher levels of combined (moderate + strenuous), lifetime PA (42.9%) and lower levels of combined, recent PA (37.3%). While current smokers were more likely to have lower levels of combined, recent PA (43.3%), former smokers were more likely to have higher levels of combined, recent PA (37.2%). Women with a BMI ≥ 30 kg/m^3^ had lower levels of combined, recent PA (51%).

**Table 1:** Baseline participant characteristics among 1,768 women diagnosed with lung cancer in the California Teachers Study stratified by pre-diagnosis physical activity levels (presented as tertiles)

		Lifetime physical activity^a^			Recent physical activity^a,b^		
Characteristic	N(%)	Low	Intermediate	High	Low	Intermediate	High
No. of invasive lung cancer cases	1768	590	591	587	598	589	581
Mean age at diagnosis ± SD		75.6 ± 9.4	74.4 ± 9.7	74.1 ± 10.5	74.7 ± 10	74.1 ± 9.8	75.2 ± 9.8
*Race/ethnicity (%)*							
Non-Hispanic White	1569(88.7%)	33.1	33.1	33.8	32.4	33.8	33.8
Others^c^	199(11.3%)	35.2	36.2	28.6	45.2	29.7	25.1
*Socioeconomic status (%)*							
Low SES	68(3.9%)	30.9	41.2	27.9	39.7	35.3	25.0
2^nd^quartile	275(15.6%)	35.6	28.7	35.6	43.3	27.3	29.5
3^rd^quartile	515(29.1%)	36.3	31.8	31.8	35.3	32.2	32.4
High SES	900(50.9%)	31.2	34.9	33.9	29.8	35.3	34.9
*Menopausal status (%)*							
Premenopausal	177(10%)	22.0	35.0	42.9	37.3	33.9	28.8
*Postmenopausal*							
No hormone therapy use	391(22.1%)	34.0	32.5	33.5	35.3	31.0	33.8
Current/former hormone therapy use	975(55.2%)	34.8	33.5	31.7	32.1	33.0	34.9
All Others	225(12.7%)	35.1	33.3	31.6	36.0	38.2	25.8
*Smoking status(%)*							
Never smoker	516(29.2%)	36.1	31.4	32.6	32.2	36.8	31.0
Former smoker	822(46.5%)	32.5	35.3	32.2	29.9	32.9	37.2
Current smoker	430(24.3%)	31.9	32.3	35.8	43.3	30.0	26.7
Mean pack-years ± SD		35.8 ± 24.3	30.9 ± 21.9	32.9 ± 22.5	37.9 ± 25.6	30.8 ± 21.7	30.5 ± 20.5
*Body Mass Index, kg/m*^*2*^*(%)*							
< 18	129(7.3%)	42.6	30.2	27.1	37.2	34.9	27.9
18–24	963(54.5%)	32.6	34.4	33.0	28.7	33.0	38.3
25–30	476(26.9%)	31.7	33.6	34.7	35.9	35.1	29.0
≥ 30	200(11.3%)	35.0	30.5	34.5	51.5	29.5	19.0
*Cancer Histology(%)*							
Non-small cell lung cancer	1647(93.2%)	33.0	33.9	33.1	33.2	33.5	33.3
Small cell lung cancer	121(6.8%)	38.0	27.3	34.7	43.0	30.6	26.5
*Stage (%)*							
Localized	455(25.7%)	28.8	36.9	34.3	32.3	35.2	32.5
Regional extension only	123(7%)	30.9	37.4	31.7	29.3	33.3	37.4
Regional lymph nodes only	134(7.6%)	37.3	31.3	31.3	38.1	35.1	26.9
Regional extension and lymph nodes	87(4.9%)	31.0	29.9	39.1	32.2	29.9	37.9
Distant	879(49.7%)	35.4	31.4	33.2	33.9	32.9	33.2

^a^Lifetime and recent categories include physical activity presented as tertiles

^b^Recent includes PA reported within 3 years prior to baseline

^c^The “Other” category for race/ethnicity includes Hispanic, Native American, Asian/Pacific Islander, Mixed, and Unknown

**Fig. 1:**
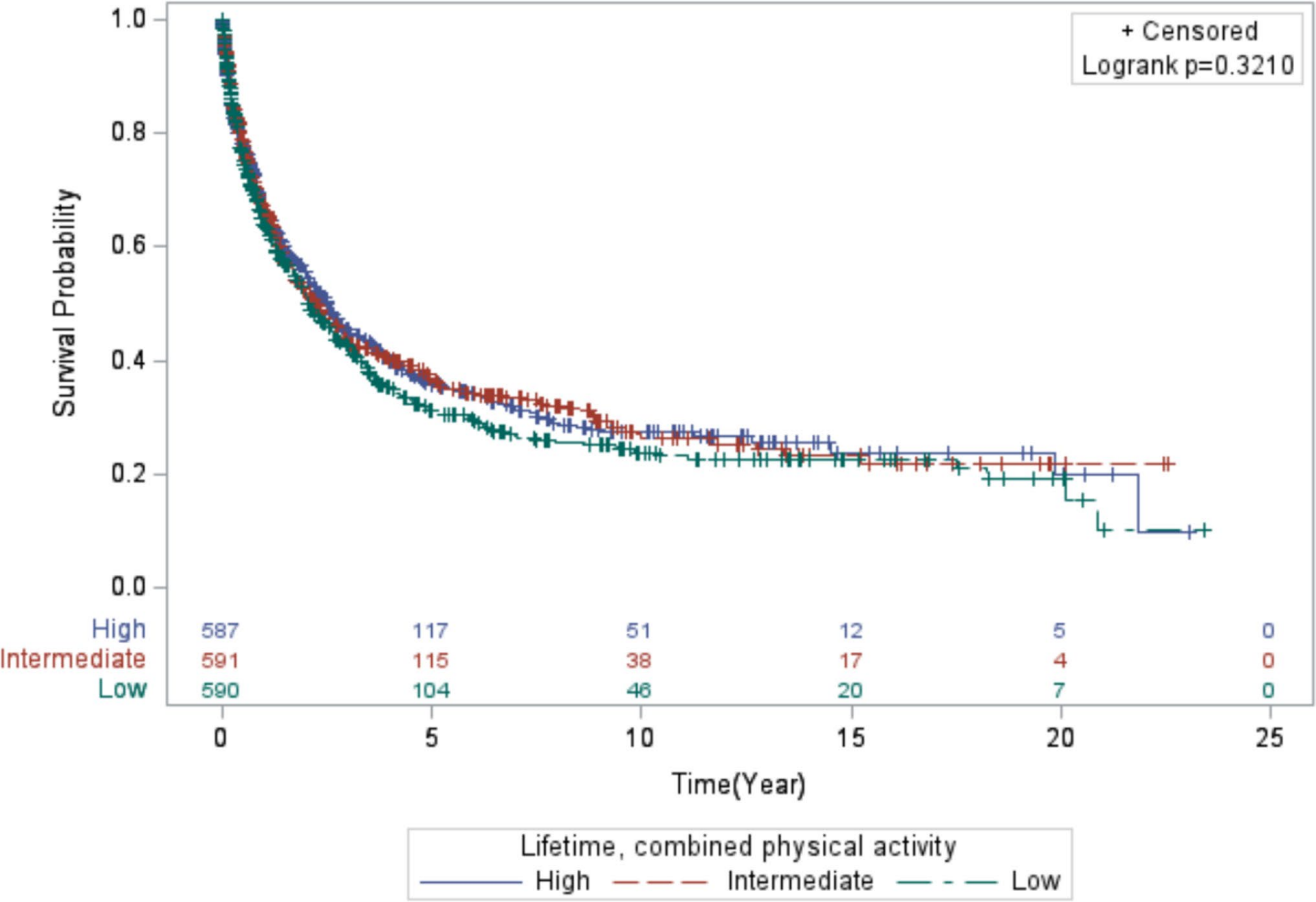
Kaplan–Meier curve displaying the survival time of the women in the study who engaged in lifetime, combined physical activity

**Fig. 2:**
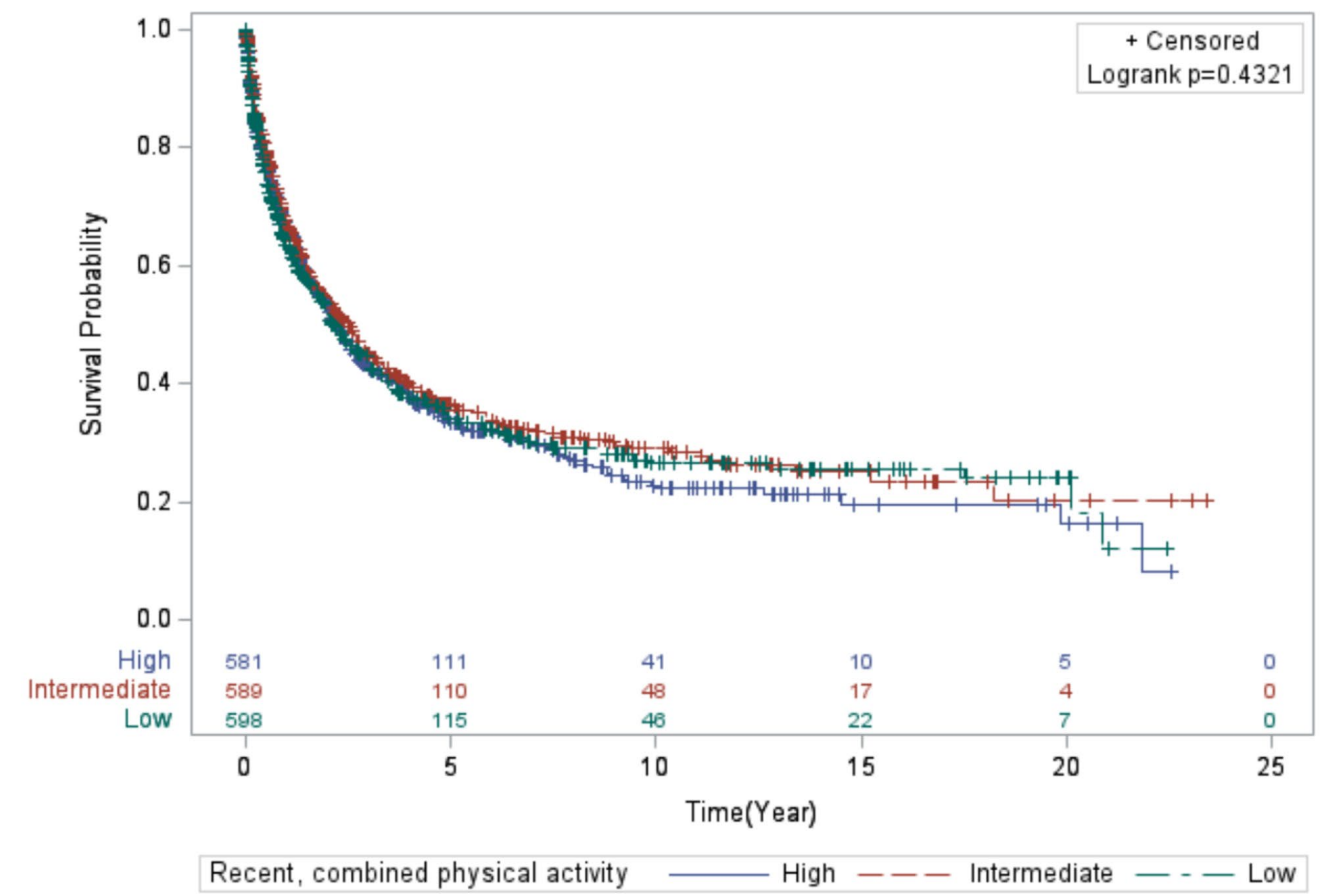
Kaplan–Meier curve displaying the survival time of the women in the study who engaged in recent, combined physical activity

The results (Model 1 and Model 2) for the associations between all PA variables and lung cancer mortality are presented in Table 2. Overall, the results for lung cancer mortality were relatively unremarkable across all PA variables for lifetime and recent activity. Women who reported higher levels of combined, lifetime PA had the highest median survival time (MST) of 2.5 years compared to 2.1 years for women who reported lower activity levels and 2.3 years for women who reported intermediate levels (*Log-rank p value*: 0.321; Fig. 1). In contrast, for combined, recent PA, those with intermediate levels of activity survived longer (MST = 2.5 years) compared to those with lower (MST = 2.2) and higher combined, recent PA levels (MST = 2.2 years) (*Log-rank p value*: 0.4321; Fig. 2).

**Table 2:** Multivariable hazard ratios (HR) and 95% confidence intervals (CI) for the association between physical activity (measured in tertiles) and mortality among 1,768 women diagnosed with invasive lung cancer following enrollment in the California Teachers Study

	N Cases	N Deaths	Model 1^a^	Model 2^b^
			HR (95% CI)	HR (95% CI)
Lifetime physical activity				
Moderate				
Low	611	370	1.00 (Reference)	1.00 (Reference)
Intermediate	571	331	0.96 (0.82–1.11)	0.96 (0.82–1.12)
High	586	342	0.87 (0.74–1.03)	0.87 (0.73–1.03)
P for trend			0.1017	0.1078
Strenuous				
Low	590	353	1.00 (Reference)	1.00 (Reference)
Intermediate	589	343	1.01 (0.87–1.17)	1.10 (0.94–1.28)
High	589	347	1.00 (0.84–1.18)	1.07 (0.90–1.26)
P for trend			0.9682	0.3977
Moderate + Strenuous				
Low	590	360	1.00 (Reference)	1.00 (Reference)
Intermediate	591	339	0.95 (0.82–1.11)	0.96 (0.83–1.12)
High	587	344	0.93 (0.80–1.08)	0.97 (0.83–1.13)
P for trend			0.3187	0.6778
Recent physical activity				
Moderate				
Low	630	362	1.00 (Reference)	1.00 (Reference)
Intermediate	688	402	1.04 (0.90–1.20)	1.06 (0.92–1.23)
High	450	279	1.08 (0.91–1.27)	1.06 (0.90–1.25)
P for trend			0.3896	0.4760
Strenuous				
Low	896	539	1.00 (Reference)	1.00 (Reference)
Intermediate	322	189	1.17 (0.99–1.39)	1.11 (0.94–1.32)
High	550	315	0.93 (0.80–1.08)	0.98 (0.85–1.14)
P for trend			0.4861	0.9595
Moderate + Strenuous				
Low	598	350	1.00 (Reference)	1.00 (Reference)
Intermediate	589	337	0.96 (0.83–1.12)	1.03 (0.89–1.21)
High	581	356	1.03 (0.88–1.19)	1.07 (0.92–1.24)
P for trend			0.7471	0.4143

^a^Model 1: adjusted for age at lung cancer diagnosis and the other respective PA variables

^b^Model 2: adjusted for factors in Model 1 and menopause status with hormonal therapy use, smoking total pack-years by smoking status, and alcohol consumption

**Fig. 3:**
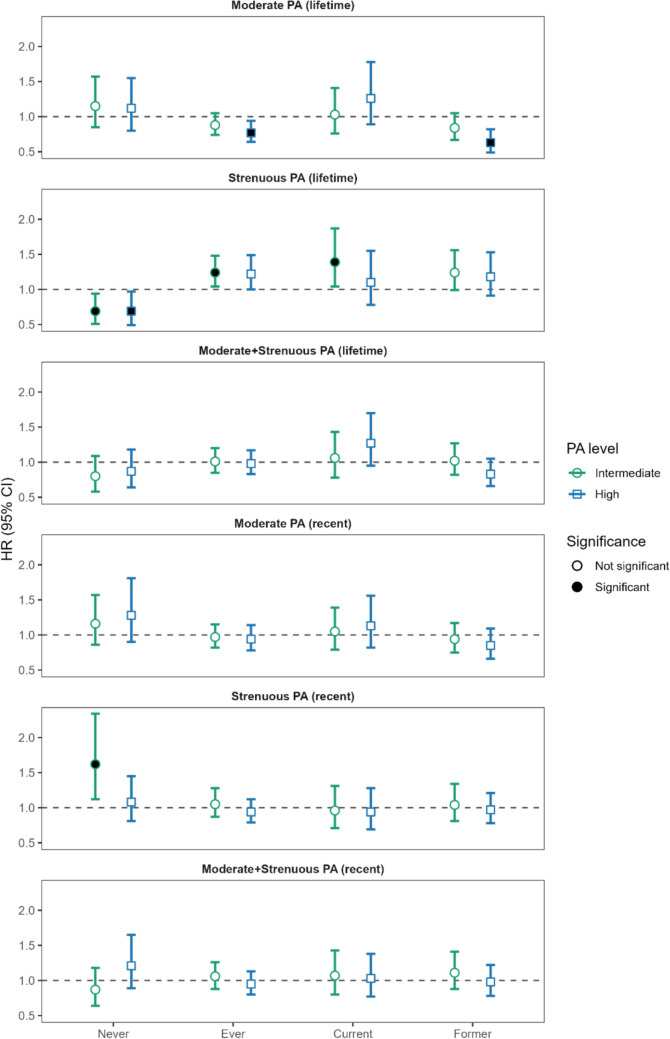
Multivariable hazard ratios (HR) and 95% confidence intervals (CI) for the association between physical activity and mortality among 1,768 women diagnosed with invasive lung cancer following enrollment in the California Teachers Study stratified by smoking status and adjusted for age at lung cancer diagnosis, the other respective PA variables, menopause status with hormonal therapy, and alcohol consumption

Associations stratified by smoking status are presented in Fig. 3 and Supplement Table S2. Ever smokers (HR = 0.77, 95%CI = 0.64–0.94) and former smokers (HR = 0.63, 95%CI = 0.49–0.82) who engaged in higher levels of moderate, lifetime PA had a decreased risk of lung cancer mortality. Ever smokers who engaged in intermediate (HR = 1.24, 95%CI = 1.04–1.48) and higher (HR = 1.22, 95%CI = 1.00–1.49) levels of strenuous, lifetime PA saw increased risk of lung cancer mortality. Similarly, current smokers who engaged in intermediate levels of strenuous, lifetime PA also saw increased risk of lung cancer mortality (HR = 1.39, 95%CI = 1.04–1.87). In contrast, never smokers who engaged in higher levels of strenuous, lifetime PA saw a decrease in lung cancer mortality risk (HR = 0.69, 95%CI = 0.49–0.97). Additionally, never smokers saw an increased risk of lung cancer mortality with intermediate levels of strenuous, recent PA (HR = 1.62, 95%CI = 1.12–2.34).

Sensitivity analyses were performed by adjusting for BMI, education level, and passive smoke exposure; the results remain consistent with the main models (Supplemental Table S3). We additionally conducted a sensitivity analysis excluding lung cancer-specific deaths within the first 5 years of follow‑up to reduce the influence of preclinical disease and early mortality, which can exacerbate collider mechanisms in stratified analyses (Supplemental Table S4). The results were relatively consistent with the main models, except that the increased risks observed among ever and/or current smokers were no longer statistically significant. We note that the direction and magnitude of these associations were similar to the primary findings, and the loss of statistical significance is likely due to reduced sample size and fewer events after removing early deaths. Further adjustment by smoking quit-years for former smokers yielded similar results to the main smoking stratified results (Supplemental Table S5). Inclusion of disease stage in the models (Supplemental Table S6) produced patterns that were largely consistent with the main results. Among former smokers, higher levels of moderate, lifetime PA remained protective, although the association was no longer statistically significant. Among ever smokers, the intermediate and high levels of moderate, lifetime PA were essentially null. The direction of associations among ever and current smokers engaging in intermediate to high strenuous, lifetime PA continued to indicate higher mortality risk, while never smokers, again, demonstrated lower risk at higher levels of strenuous, lifetime PA. Never smokers engaging in intermediate levels of strenuous, recent PA also continued to show increased mortality risk, which mirrors the main models. The inclusion of disease stage attenuated some estimates but did not materially change the overall pattern of associations, suggesting that our findings are not solely driven by differences in stage at diagnosis.

## Discussion

We assessed associations between PA and lung cancer-specific survival among 1,768 women diagnosed with lung cancer and enrolled in the California Teachers Study from 1995–2019. We highlight the following notable associations from this study: (1) women who have smoked (ever and/or former) and engaged in higher levels of moderate, lifetime PA tended to have lower lung cancer-specific mortality, (2) ever and/or current smokers who engaged in intermediate to high levels of strenuous, lifetime PA appeared to have higher mortality risk, while never smokers showed evidence of lower mortality with higher levels of strenuous, lifetime PA, and (3) never smokers who engaged in intermediate levels of strenuous, recent PA exhibited a suggested increase in lung cancer-specific mortality. However, these stratified associations should be interpreted with caution. Many of the smoking‑stratified estimates were based on relatively small numbers and were characterized by wide confidence intervals, particularly among current and former smokers. These estimates limit the ability to draw firm conclusions about whether strenuous physical activity is harmful or beneficial for individuals with a smoking history. Our results should therefore be viewed as exploratory.

While a relatively small number of studies have examined PA and lung cancer-specific mortality, fewer still have explored this association while also stratifying by smoking status, a key determinant of both incidence and mortality in lung cancer. Among those that do not stratify by smoking status, increased PA levels were consistently associated with reduced lung cancer mortality [14, 20]. We found only three studies to date that examine PA and risk of lung cancer mortality across smoke exposure groups in women [15, 26]. However, these studies either assess recent PA only or do not account for both duration and exertion of PA, limiting the comparability of their findings to those of the present study.

PA is known to support immune cell infiltration, reduce cancer cell growth and survival, improve the tumor microenvironment, reduce oxidative stress and increase DNA repair processes [27]. Despite these benefits, several studies suggest that PA’s therapeutic effects persist to an extent, while excessive strenuous PA may have an opposite and deleterious effect on lung cancer mortality [16, 19, 28]. Exercise immunology research indicates that high-intensity PA without adequate recovery can trigger immunosuppressive effects that temporarily increase the risk of illness [29, 30]. Specifically, it is thought that following strenuous PA, antibody production as well as circulating levels of lymphocytes and natural killer cells significantly decreases [29, 31, 32]. While more research is warranted to confirm our findings, this temporary immunosuppressive state could help conceptualize the increased risk of lung cancer mortality we observed among never-smoking women who engaged in strenuous, recent PA. While more research is needed, certain exercise immunology findings are consistent with the patterns observed. Specifically, high‑intensity physical activity without sufficient recovery has been associated with transient reductions in immune function, which could plausibly influence post‑diagnosis outcomes, particularly among individuals already experiencing chronic inflammation from smoking [9]. These mechanistic considerations, however, remain speculative and cannot be confirmed within this study.

Moreover, former and ever smokers who engaged in higher levels of moderate lifetime PA showed evidence of more favorable survival while higher strenuous PA levels did not show similar patterns, potentially highlighting the importance of quality and duration spent in PA recovery. Moderate lifetime PA, such as brisk walking or playing golf, may provide conditions supportive of cell recovery and immune repair necessary to mitigate the deleterious effects of smoking, but this interpretation remains tentative and will require confirmation in future, larger studies [9, 15].

The limited research on this topic has largely focused on recent, pre-lung cancer diagnosis PA. Interestingly, our stratified analyses revealed a stark contrast in lung cancer mortality risks between lifetime PA and recent PA groups, suggesting that PA and smoking behaviors from high school through adulthood may play a more prominent role in women’s lung cancer survival than in the several years preceding diagnosis. Further studies examining lifetime and exertion-varying PA variables and lung cancer mortality are warranted.

Our findings ultimately expand on those of previous studies, though several unexpected outcomes reveal the need for additional research in this area. We draw the following conclusions about PA and lung cancer mortality prevention, all of which should be verified in future studies: (1) PA regimens (e.g., PA intensity and duration) may benefit from a more personalized approach, taking into account factors like disease stage, physical fitness, smoking status, and other health behaviors; (2) PA interventions aimed at lung cancer survival improvement may be most effective if initiated early, as suggested by previous work [18]; and (3) interventions and public/clinical health promotion efforts may benefit from considering the possibility that high levels of strenuous PA among those with a history of smoking may not provide the same benefits as moderate PA.

The strengths of this study include its prospective design, recent and length of follow-up time (up to 2019), detailed lifetime and recent PA information categorized by exertion level, and extensive data regarding potential confounding factors (e.g., total smoking pack-years and passive household smoke exposure). One limitation of this study is the timing of PA measures. The data were collected at baseline, with a varying number of years preceding diagnosis; thus, recent PA accounted for the three years prior to enrollment, not diagnosis. Another limitation is the potential for collider bias due to smoking status stratification, as mortality is jointly influenced by smoking, physical activity, and other health factors [33]. Because this approach could distort the associations between PA and lung cancer mortality within smoking groups, the stratified results should be interpreted with caution. We did, however, conduct a sensitivity analysis that excluded lung cancer-specific deaths within the first 5 years of follow-up. Results of these analyses were similar to the main findings, suggesting that collider bias is unlikely to fully account for the observed associations. Additional limitations include (1) MET-hours of PA are a more accurate way to measure intensity and duration of PA; however, detailed information regarding specific types of PA performed during each time period (e.g., walking vs cycling), which would be needed to calculate MET-hours/week, was not collected in the baseline CTS questionnaire; (2) the smoking variables included in this study were collected at baseline, are restricted to cigarette smoking and do not reflect smoking cessation during follow-up; (3) our study was based in California, a state reported to have high pollution levels, which have previously been associated with lung cancer risk [34, 35]; (4) our study population is restricted to women only, potentially reducing generalizability; however, postmenopausal women are seldom the focus of studies on lung cancer mortality and PA levels, despite lung cancer remaining the second most common cancer in women, secondary to breast cancer [1]; and (5) because the majority of participants identified as non‑Hispanic White, the extent to which these findings generalize to more diverse racial and ethnic populations may be limited. Given the potential for clinical significance if our findings are confirmed in future studies, further investigation is warranted.

## Supplementary Information

Below is the link to the electronic supplementary material.

Supplementary file1 (DOCX 42 kb)

## Data Availability

The California Teachers Study data and resources are made available in accordance with the National Institute of Health’s Policy for Data Management and Sharing and the NIH Genomic Data Sharing Policy. The CTS Data Environment, which includes all CTS data, software, and documentation, is open and free of charge to anyone who agrees to and signs the CTS Confidentiality Pledge. Individuals interested in accessing CTS data can sign up for the CTS Researcher Platform and submit a project for feasibility review here: (https://calteachersstudy.my.site.com/researchers/s/). The dataset generated and analyzed for the current study is not publicly available as it is housed within the CTS Data Environment but can be made available by the corresponding author on reasonable request.
