# Supplementary material for: Pre-diagnosis recreational physical activity and lung cancer survival within the California teachers study
Source: Cancer Causes Control. 2026 Apr 22;37(6):87. doi: 10.1007/s10552-026-02169-6 (PMC13102877; doi:10.1007/s10552-026-02169-6)
Supplement: Supplementary file 1 — Supplementary file1 (DOCX 42 kb) [file 10552_2026_2169_MOESM1_ESM.docx]

**Pre-Diagnosis Recreational Physical Activity and Lung Cancer Survival within the California Teachers Study**

Emily L. Cauble, Mia Blanchard, Peggy Reynolds, Emma S. Spielfogel, Jessica Clague DeHart

**Corresponding author:**

Jessica Clague DeHart, PhD, MPH

Claremont Graduate University, School of Community and Global Health, Claremont, CA, USA

[Jessica.dehart@cgu.edu](mailto:Jessica.dehart@cgu.edu)

*Cancer Causes and Control*

**Supplemental Table S1.** Descriptive statistics (hours/week/year) for the physical activity (PA) variables (lifetime= high school through age 54 years; recent=within 3 years of complete the baseline questionnaire) among 1,786 women diagnosed with lung cancer in the California Teachers Study.

| **PA Variables** | **N** | **Mean** | **Standard deviation** | **33rd Percentile^a^** | **Median** | **67th Percentile^a^** | **Maximum** |
| --- | --- | --- | --- | --- | --- | --- | --- |
| Moderate, lifetime | 1768 | 2.35 | 2.38 | 1.00 | 1.65 | 2.56 | 12.00 |
| Strenuous, lifetime | 1768 | 1.77 | 2.13 | 0.44 | 1.01 | 1.92 | 12.00 |
| Combined, lifetime | 1768 | 4.12 | 3.96 | 1.85 | 2.95 | 4.56 | 24.00 |
| Moderate, recent | 1768 | 2.47 | 2.77 | 0.75 | 1.50 | 3.00 | 12.00 |
| Strenuous, recent | 1768 | 1.31 | 2.22 | 0.00 | 0.00 | 1.00 | 12.00 |
| Combined, recent | 1768 | 3.78 | 4.19 | 1.13 | 2.50 | 4.50 | 24.00 |
| ^a^Tertiles for PA variables were determined by the 33.33rd and 66.67th percentiles | | | | | | | |

**Supplemental Table S2.** Multivariable hazard ratios (HR) and 95% confidence intervals (CI) for the associations between physical activity and mortality among 1,768 women diagnosed with invasive lung cancer following enrollment in the California Teachers Study stratified by smoking status.

|  | **Never Smokers** | | | **Ever Smokers** | | | **Former Smokers** | | | **Current Smokers** | | |
| --- | --- | --- | --- | --- | --- | --- | --- | --- | --- | --- | --- | --- |
|  | **N** | **Model 1^a^** | **Model 2^b^** | **N** | **Model 1^a^** | **Model 2^b^** | **N** | **Model 1^a^** | **Model 2^b^** | **N** | **Model 1^a^** | **Model 2^b^** |
|  | **Deaths/Cases** | **HR (95% CI)** | **HR (95% CI)** | **Deaths/Cases** | **HR (95% CI)** | **HR (95% CI)** | **Deaths/Cases** | **HR (95% CI)** | **HR (95% CI)** | **Deaths/Cases** | **HR (95% CI)** | **HR (95% CI)** |
| **Lifetime PA** |  |  |  |  |  |  |  |  |  |  |  |  |
| **Moderate** |  |  |  |  |  |  |  |  |  |  |  |  |
| Low | 91/188 | 1.00 (Reference) | 1.00 (Reference) | 279/423 | 1.00 (Reference) | 1.00 (Reference) | 175/282 | 1.00 (Reference) | 1.00 (Reference) | 104/141 | 1.00 (Reference) | 1.00 (Reference) |
| Intermediate | 85/163 | 1.16 (0.86-1.57) | 1.15 (0.85-1.57) | 246/408 | 0.87 (0.72-1.03) | 0.88 (0.74-1.05) | 162/272 | 0.86 (0.69-1.08) | 0.84 (0.67-1.05) | 84/136 | 0.86 (0.64-1.16) | 1.03 (0.76-1.41) |
| High | 87/165 | 1.11 (0.80-1.54) | 1.12 (0.80-1.55) | 255/421 | 0.76 (0.62-0.92) | 0.77 (0.64-0.94) | 146/268 | 0.64 (0.50-0.82) | 0.63 (0.49-0.82) | 109/153 | 1.09 (0.78-1.54) | 1.26 (0.89-1.78) |
| *P for trend* |  | 0.4805 | 0.482 |  | 0.0058 | 0.0105 |  | 0.0005 | 0.0004 |  | 0.6768 | 0.2196 |
| **Strenuous** |  |  |  |  |  |  |  |  |  |  |  |  |
| Low | 99/179 | 1.00 (Reference) | 1.00 (Reference) | 254/411 | 1.00 (Reference) | 1.00 (Reference) | 155/269 | 1.00 (Reference) | 1.00 (Reference) | 99/142 | 1.00 (Reference) | 1.00 (Reference) |
| Intermediate | 81/171 | 0.73 (0.54-0.98) | 0.69 (0.51-0.94) | 271/418 | 1.18 (0.99-1.41) | 1.24 (1.04-1.48) | 173/286 | 1.16 (0.93-1.46) | 1.24 (0.99-1.56) | 98/132 | 1.31 (0.98-1.75) | 1.39 (1.04-1.87) |
| High | 83/166 | 0.71 (0.51-0.99) | 0.69 (0.49-0.97) | 264/423 | 1.17 (0.96-1.42) | 1.22 (1.00-1.49) | 155/267 | 1.15 (0.90-1.48) | 1.18 (0.91-1.53) | 109/156 | 1.16 (0.83-1.62) | 1.10 (0.78-1.55) |
| *P for trend* |  | 0.0327 | 0.024 |  | 0.0898 | 0.0312 |  | 0.2434 | 0.1668 |  | 0.2755 | 0.3603 |
| **Moderate + Strenuous** |  |  |  |  |  |  |  |  |  |  |  |  |
| Low | 97/186 | 1.00 (Reference) | 1.00 (Reference) | 263/404 | 1.00 (Reference) | 1.00 (Reference) | 164/267 | 1.00 (Reference) | 1.00 (Reference) | 99/137 | 1.00 (Reference) | 1.00 (Reference) |
| Intermediate | 78/162 | 0.89 (0.66-1.20) | 0.80 (0.58-1.09) | 261/429 | 0.98 (0.82-1.16) | 1.01 (0.85-1.20) | 170/290 | 1.00 (0.80-1.24) | 1.02 (0.82-1.27) | 91/139 | 0.98 (0.74-1.32) | 1.06 (0.78-1.43) |
| High | 88/168 | 0.89 (0.66-1.19) | 0.87 (0.64-1.18) | 256/419 | 0.94 (0.79-1.12) | 0.98 (0.83-1.17) | 149/265 | 0.83 (0.67-1.04) | 0.83 (0.66-1.05) | 107/154 | 1.24 (0.94-1.64) | 1.27 (0.95-1.70) |
| *P for trend* |  | 0.4231 | 0.3792 |  | 0.4897 | 0.8489 |  | 0.1139 | 0.1217 |  | 0.1282 | 0.1038 |
| **Recent PA** |  |  |  |  |  |  |  |  |  |  |  |  |
| **Moderate** |  |  |  |  |  |  |  |  |  |  |  |  |
| Low | 81/179 | 1.00 (Reference) | 1.00 (Reference) | 281/451 | 1.00 (Reference) | 1.00 (Reference) | 152/266 | 1.00 (Reference) | 1.00 (Reference) | 129/185 | 1.00 (Reference) | 1.00 (Reference) |
| Intermediate | 116/221 | 1.27 (0.94-1.70) | 1.16 (0.86-1.57) | 286/467 | 0.97 (0.82-1.15) | 0.97 (0.82-1.15) | 190/321 | 1.00 (0.80-1.25) | 0.94 (0.75-1.17) | 96/146 | 0.98 (0.75-1.29) | 1.05 (0.79-1.39) |
| High | 66/116 | 1.43 (1.02-2.02) | 1.28 (0.90-1.81) | 213/334 | 0.93 (0.76-1.12) | 0.94 (0.78-1.14) | 141/235 | 0.89 (0.69-1.14) | 0.85 (0.66-1.09) | 72/99 | 1.09 (0.79-1.50) | 1.13 (0.82-1.56) |
| *P for trend* |  | 0.0338 | 0.1564 |  | 0.4355 | 0.5457 |  | 0.3568 | 0.1865 |  | 0.6506 | 0.4713 |
| **Strenuous** |  |  |  |  |  |  |  |  |  |  |  |  |
| Low | 127/253 | 1.00 (Reference) | 1.00 (Reference) | 412/643 | 1.00 (Reference) | 1.00 (Reference) | 250/416 | 1.00 (Reference) | 1.00 (Reference) | 162/227 | 1.00 (Reference) | 1.00 (Reference) |
| Intermediate | 46/90 | 1.48 (1.04-2.11) | 1.62 (1.12-2.34) | 143/232 | 1.03 (0.85-1.25) | 1.05 (0.87-1.28) | 80/136 | 1.05 (0.81-1.35) | 1.04 (0.81-1.34) | 63/96 | 0.96 (0.71-1.29) | 0.96 (0.71-1.31) |
| High | 90/173 | 1.03 (0.78-1.38) | 1.08 (0.81-1.45) | 225/377 | 0.93 (0.78-1.10) | 0.94 (0.79-1.12) | 153/270 | 0.99 (0.79-1.22) | 0.97 (0.78-1.21) | 72/107 | 0.89 (0.66-1.20) | 0.94 (0.69-1.28) |
| *P for trend* |  | 0.7206 | 0.54 |  | 0.4619 | 0.5988 |  | 0.9365 | 0.862 |  | 0.4323 | 0.6581 |
| **Moderate + Strenuous** |  |  |  |  |  |  |  |  |  |  |  |  |
| Low | 80/166 | 1.00 (Reference) | 1.00 (Reference) | 270/432 | 1.00 (Reference) | 1.00 (Reference) | 140/246 | 1.00 (Reference) | 1.00 (Reference) | 130/186 | 1.00 (Reference) | 1.00 (Reference) |
| Intermediate | 90/190 | 0.91 (0.67-1.23) | 0.87 (0.64-1.18) | 247/399 | 1.06 (0.89-1.26) | 1.06 (0.88-1.26) | 161/270 | 1.15 (0.92-1.45) | 1.11 (0.88-1.41) | 86/129 | 1.01 (0.77-1.34) | 1.07 (0.80-1.43) |
| High | 93/160 | 1.31 (0.97-1.78) | 1.21 (0.89-1.65) | 263/421 | 0.94 (0.79-1.11) | 0.95 (0.80-1.13) | 182/306 | 1.04 (0.83-1.30) | 0.98 (0.78-1.22) | 81/115 | 0.95 (0.71-1.26) | 1.03 (0.77-1.38) |
| *P for trend* |  | 0.0862 | 0.2175 |  | 0.4535 | 0.5381 |  | 0.8168 | 0.7714 |  | 0.72 | 0.8137 |
| ^a^Model 1: adjusted for age at lung cancer diagnosis and the other respective PA variables  ^b^Model 2: adjusted for factors in Model 1 and menopause status with hormonal therapy use, and alcohol consumption | | | | | | | | | | | | |

**Supplemental Table S3.** Hazard ratios (HRs) and 95% confidence intervals (CIs) from sensitivity analyses evaluating potential confounding by body mass index (BMI), education level, and passive smoking exposure in the associations between physical activity and lung cancer-specific mortality among 1,768 women diagnosed with invasive lung cancer in the California Teachers Study. Results from the primary Model 2 (as presented in Table 2) are shown alongside Model 2 with the additional covariate adjustments.

|  |  | **Model 2 from original models (same result presented in Table 2)** | **Model 2 with additional adjustment for BMI, education level, and passive smoking status** |
| --- | --- | --- | --- |
|  |  | **HR (95% CI)** | **HR (95% CI)** |
| **Moderate lifetime PA** | Reference | 1.00 (Reference) | 1.00 (Reference) |
|  | Intermediate | 0.96 (0.82-1.12) | 0.96 (0.82-1.12) |
|  | High | 0.87 (0.73-1.03) | 0.87 (0.74-1.04) |
| **Strenuous lifetime PA** | Reference | 1.00 (Reference) | 1.00 (Reference) |
|  | Intermediate | 1.10 (0.94-1.28) | 1.11 (0.95-1.29) |
|  | High | 1.07 (0.90-1.26) | 1.07 (0.90-1.27) |
| **Moderate + Strenuous lifetime PA** | Reference | 1.00 (Reference) | 1.00 (Reference) |
|  | Intermediate | 0.96 (0.83-1.12) | 0.97 (0.83-1.12) |
|  | High | 0.97 (0.83-1.13) | 0.95 (0.81-1.10) |
| **Moderate recent PA** | Reference | 1.00 (Reference) | 1.00 (Reference) |
|  | Intermediate | 1.06 (0.92-1.23) | 1.09 (0.94-1.27) |
|  | High | 1.06 (0.90-1.25) | 1.06 (0.90-1.26) |
| **Strenuous recent PA** | Reference | 1.00 (Reference) | 1.00 (Reference) |
|  | Intermediate | 1.11 (0.94-1.32) | 1.13 (0.96-1.35) |
|  | High | 0.98 (0.85-1.14) | 0.98 (0.84-1.13) |
| **Moderate + Strenuous recent PA** | Reference | 1.00 (Reference) | 1.00 (Reference) |
|  | Intermediate | 1.03 (0.89-1.21) | 1.08 (0.92-1.26) |
|  | High | 1.07 (0.92-1.24) | 1.07 (0.92-1.25) |

**Supplemental Table S4.** Sensitivity analysis excluding lung cancer deaths occurring within the first 5 years of follow‑up to reduce potential reverse causation and early mortality bias. Hazard ratios (HRs) and 95% confidence intervals (CIs) for the associations between physical activity and lung cancer-specific mortality are presented for 1,491 women diagnosed with invasive lung cancer in the California Teachers Study (fatal cases = 878; non‑fatal cases = 613), stratified by smoking status.

|  |  | **Never**  **(fatal N=224)** | **Ever**  **(fatal N=654)** | **Former**  **(fatal N=418)** | **Current**  **(fatal N=236)** |
| --- | --- | --- | --- | --- | --- |
|  |  | **HR (95% CI)** | **HR (95% CI)** | **HR (95% CI)** | **HR (95% CI)** |
| **Moderate lifetime PA** | Reference | 1.00 (Reference) | 1.00 (Reference) | 1.00 (Reference) | 1.00 (Reference) |
|  | Intermediate | 1.10 (0.79-1.54) | 0.89 (0.74-1.08) | 0.84 (0.66-1.07) | 0.98 (0.69-1.39) |
|  | High | 1.18 (0.84-1.68) | 0.80 (0.64-0.99) | 0.61 (0.46-0.80) | 1.44 (0.95-2.16) |
| **Strenuous lifetime PA** | Reference | 1.00 (Reference) | 1.00 (Reference) | 1.00 (Reference) | 1.00 (Reference) |
|  | Intermediate | 0.66 (0.47-0.92) | 1.19 (0.98-1.44) | 1.21 (0.94-1.54) | 1.39 (0.99-1.94) |
|  | High | 0.69 (0.47-0.99) | 1.17 (0.94-1.45) | 1.20 (0.91-1.58) | 0.93 (0.63-1.38) |
| **Moderate + Strenuous lifetime PA** | Reference | 1.00 (Reference) | 1.00 (Reference) | 1.00 (Reference) | 1.00 (Reference) |
|  | Intermediate | 0.73 (0.52-1.04) | 1.02 (0.85-1.24) | 1.03 (0.81-1.30) | 0.97 (0.69-1.38) |
|  | High | 0.91 (0.65-1.28) | 0.96 (0.79-1.16) | 0.81 (0.63-1.04) | 1.16 (0.83-1.61) |
| **Moderate recent PA** | Reference | 1.00 (Reference) | 1.00 (Reference) | 1.00 (Reference) | 1.00 (Reference) |
|  | Intermediate | 1.13 (0.82-1.57) | 1.00 (0.83-1.21) | 0.93 (0.73-1.19) | 1.05 (0.76-1.45) |
|  | High | 1.50 (1.03-2.17) | 0.96 (0.78-1.19) | 0.83 (0.64-1.10) | 1.03 (0.72-1.47) |
| **Strenuous recent PA** | Reference | 1.00 (Reference) | 1.00 (Reference) | 1.00 (Reference) | 1.00 (Reference) |
|  | Intermediate | 1.65 (1.11-2.46) | 1.05 (0.85-1.31) | 1.06 (0.80-1.39) | 0.96 (0.66-1.38) |
|  | High | 1.12 (0.82-1.53) | 0.92 (0.76-1.11) | 0.97 (0.77-1.23) | 0.92 (0.65-1.28) |
| **Moderate + Strenuous recent PA** | Reference | 1.00 (Reference) | 1.00 (Reference) | 1.00 (Reference) | 1.00 (Reference) |
|  | Intermediate | 0.79 (0.57-1.11) | 1.12 (0.93-1.36) | 1.12 (0.87-1.43) | 1.14 (0.81-1.60) |
|  | High | 1.28 (0.92-1.79) | 0.98 (0.81-1.18) | 1.00 (0.78-1.27) | 0.94 (0.67-1.31) |

**Supplemental Table S5.** Sensitivity analysis among former smokers evaluating the potential influence of smoking quit‑years on the associations between physical activity and lung cancer-specific mortality. Hazard ratios (HRs) and 95% confidence intervals (CIs) are presented for former smokers using the primary Model 2 (as shown in Supplemental Table S2) alongside Model 2 additionally adjusted for years since quitting smoking.

|  |  | **Former smokers Model 2 from original models (same result presented in Supplemental Table S2)** | **Former smokers Model 2 with additional adjustment for quit-years** |
| --- | --- | --- | --- |
|  |  | **HR (95% CI)** | **HR (95% CI)** |
| **Moderate lifetime PA** | Reference | 1.00 (Reference) | 1.00 (Reference) |
|  | Intermediate | 0.84 (0.67-1.05) | 0.84 (0.67-1.06) |
|  | High | 0.63 (0.49-0.82) | 0.64 (0.50-0.83) |
| **Strenuous lifetime PA** | Reference | 1.00 (Reference) | 1.00 (Reference) |
|  | Intermediate | 1.24 (0.99-1.56) | 1.20 (0.95-1.52) |
|  | High | 1.18 (0.91-1.53) | 1.21 (0.93-1.57) |
| **Moderate + Strenuous lifetime PA** | Reference | 1.00 (Reference) | 1.00 (Reference) |
|  | Intermediate | 1.02 (0.82-1.27) | 1.05 (0.84-1.31) |
|  | High | 0.83 (0.66-1.05) | 0.84 (0.66-1.06) |
| **Moderate recent PA** | Reference | 1.00 (Reference) | 1.00 (Reference) |
|  | Intermediate | 0.94 (0.75-1.17) | 1.07 (0.85-1.35) |
|  | High | 0.85 (0.66-1.09) | 0.89 (0.69-1.16) |
| **Strenuous recent PA** | Reference | 1.00 (Reference) | 1.00 (Reference) |
|  | Intermediate | 1.04 (0.81-1.34) | 1.10 (0.84-1.43) |
|  | High | 0.97 (0.78-1.21) | 0.96 (0.76-1.21) |
| **Moderate + Strenuous recent PA** | Reference | 1.00 (Reference) | 1.00 (Reference) |
|  | Intermediate | 1.11 (0.88-1.41) | 1.22 (0.96-1.56) |
|  | High | 0.98 (0.78-1.22) | 1.05 (0.83-1.32) |

**Supplemental Table S6.** Sensitivity analysis evaluating the impact of adjusting for disease stage at diagnosis on the associations between physical activity and lung cancer-specific mortality. Hazard ratios (HRs) and 95% confidence intervals (CIs) from Model 2 additionally adjusted for disease stage are presented stratified by smoking status among 1,768 women diagnosed with invasive lung cancer in the California Teachers Study.

|  |  | **Never** | **Ever** | **Former** | **Current** |
| --- | --- | --- | --- | --- | --- |
|  |  | **HR (95% CI)** | **HR (95% CI)** | **HR (95% CI)** | **HR (95% CI)** |
| **Moderate lifetime PA** | Reference | 1.00 (Reference) | 1.00 (Reference) | 1.00 (Reference) | 1.00 (Reference) |
|  | Intermediate | 1.13 (0.83-1.54) | 0.99 (0.83-1.19) | 0.92 (0.74-1.15) | 1.16 (0.84-1.59) |
|  | High | 1.31 (0.94-1.82) | 1.01 (0.83-1.24) | 0.88 (0.68-1.14) | 1.36 (0.95-1.94) |
| **Strenuous lifetime PA** | Reference | 1.00 (Reference) | 1.00 (Reference) | 1.00 (Reference) | 1.00 (Reference) |
|  | Intermediate | 0.90 (0.66-1.23) | 1.36 (1.14-1.63) | 1.49 (1.18-1.87) | 1.31 (0.96-1.77) |
|  | High | 0.76 (0.53-1.07) | 1.17 (0.96-1.44) | 1.13 (0.87-1.46) | 1.24 (0.87-1.79) |
| **Moderate + Strenuous lifetime PA** | Reference | 1.00 (Reference) | 1.00 (Reference) | 1.00 (Reference) | 1.00 (Reference) |
|  | Intermediate | 0.88 (0.64-1.22) | 1.27 (1.07-1.52) | 1.26 (1.01-1.57) | 1.31 (0.96-1.78) |
|  | High | 0.98 (0.72-1.35) | 1.14 (0.95-1.36) | 0.96 (0.76-1.20) | 1.59 (1.18-2.13) |
| **Moderate recent PA** | Reference | 1.00 (Reference) | 1.00 (Reference) | 1.00 (Reference) | 1.00 (Reference) |
|  | Intermediate | 1.11 (0.82-1.51) | 1.00 (0.84-1.19) | 0.94 (0.75-1.18) | 1.14 (0.85-1.52) |
|  | High | 1.39 (0.97-1.98) | 0.96 (0.79-1.17) | 0.85 (0.66-1.09) | 1.25 (0.90-1.75) |
| **Strenuous recent PA** | Reference | 1.00 (Reference) | 1.00 (Reference) | 1.00 (Reference) | 1.00 (Reference) |
|  | Intermediate | 1.84 (1.26-2.67) | 1.15 (0.95-1.40) | 1.32 (1.01-1.72) | 0.89 (0.65-1.21) |
|  | High | 0.98 (0.73-1.32) | 0.93 (0.78-1.11) | 0.98 (0.78-1.22) | 0.87 (0.63-1.19) |
| **Moderate + Strenuous recent PA** | Reference | 1.00 (Reference) | 1.00 (Reference) | 1.00 (Reference) | 1.00 (Reference) |
|  | Intermediate | 0.89 (0.65-1.22) | 1.05 (0.88-1.25) | 1.13 (0.89-1.43) | 0.99 (0.74-1.33) |
|  | High | 1.15 (0.84-1.58) | 0.95 (0.80-1.13) | 0.98 (0.77-1.23) | 1.06 (0.78-1.43) |
